# A global, regional, and national survey on burden and Quality of Care Index (QCI) of brain and other central nervous system cancers; global burden of disease systematic analysis 1990-2017

**DOI:** 10.1371/journal.pone.0247120

**Published:** 2021-02-22

**Authors:** Esmaeil Mohammadi, Erfan Ghasemi, Sina Azadnajafabad, Negar Rezaei, Sahar Saeedi Moghaddam, Sepideh Ebrahimi Meimand, Nima Fattahi, Zohreh Habibi, Kourosh Karimi Yarandi, Abbas Amirjamshidi, Farideh Nejat, Farzad Kompani, Ali H. Mokdad, Bagher Larijani, Farshad Farzadfar

**Affiliations:** 1 Non-Communicable Diseases Research Center, Endocrinology and Metabolism Population Sciences Institute, Tehran University of Medical Sciences, Tehran, Iran; 2 Sina Trauma and Surgery Research Center, Tehran University of Medical Sciences, Tehran, Iran; 3 Endocrinology and Metabolism Research Center, Endocrinology and Metabolism Clinical Sciences Institute, Tehran University of Medical Sciences, Tehran, Iran; 4 Department of Neurosurgery, Children’s Hospital Medical Center, Tehran University of Medical Sciences, Tehran, Iran; 5 Department of Neurosurgery, Sina Hospital, Tehran University of Medical Sciences, Tehran, Iran; 6 Division of Hematology and Oncology, Children’s Medical Center, Pediatrics Center of Excellence, Tehran University of Medical Sciences, Tehran, Iran; 7 Institute for Health Metrics and Evaluation, University of Washington and the Department of Health Metrics Sciences, University of Washington, Seattle, WA, United States of America; Chinese Academy of Sciences, CHINA

## Abstract

Primary brain and other central nervous system (CNS) cancers cause major burdens. In this study, we introduced a measure named the Quality of Care Index (QCI), which indirectly evaluates the quality of care given to patients with this group of cancers. Here we aimed to compare different geographic and socioeconomic patterns of CNS cancer care according to the novel measure introduced. In this regard, we acquired age-standardized primary epidemiologic measures were acquired from the Global Burden of Disease (GBD) study 1990-2017. The primary measures were combined to make four secondary indices which all of them indirectly show the quality of care given to patients. Principal Component Analysis (PCA) method was utilized to calculate the essential component named QCI. Further analyses were made based on QCI to assess the quality of care globally, regionally, and nationally (with a scale of 0-100 which higher values represent better quality of care). For 2017, the global calculated QCI was 55.0. QCI showed a desirable condition in higher socio-demographic index (SDI) quintiles. Oppositely, low SDI quintile countries (7.7) had critically worse care quality. Western Pacific Region with the highest (76.9) and African Region with the lowest QCIs (9.9) were the two WHO regions extremes. Singapore was the country with the maximum QCI of 100, followed by Japan (99.9) and South Korea (98.9). In contrast, Swaziland (2.5), Lesotho (3.5), and Vanuatu (3.9) were countries with the worse condition. While the quality of care for most regions was desirable, regions with economic constraints showed to have poor quality of care and require enforcements toward this lethal diagnosis.

## Introduction

Primary brain and spinal cord tumors -collectively called central nervous system (CNS) cancers- impose a significant burden on every age group, estimated to affect 7-11 persons per 100,000 person-years [1,2]. In the latest report of the Global Burden of Disease (GBD) brain and other CNS cancers collaboration, about 330,000 new cases of CNS cancers have emerged globally. Besides, nearly 227,000 deaths and about 7.7 million Disability-Adjusted Life Years (DALYs) have been calculated because of this lethal diagnosis [2]. Being indolent and invasive added to lack of promising treatments make this entity highly disabling, peculiarly exorbitant, and not cost-beneficial for caregivers and health systems. In the last decades, the life expectancy in most of the age groups have substantially increased [3]. The extended life period combined with the higher detection rates of diseases have resulted in a higher incidence of elderly disorders, including neoplasms [4–7]. For instance, the estimated age-standardized incidence rate of CNS cancers has risen by 17.3% from 1990 to 2016 [2]. Efforts taken to elongate the onset to death time have ensued an ascent in the medical and psychological morbidities [5] in conjunction with distorted quality-of-life indices and growing expenditures [8,9] make CNS cancers a significant burden. There is no standard early detection method for brain cancers. Patients usually are diagnosed when major neurologic symptoms emerge. Most primary brain tumors present with symptoms when they become significantly large or extend to eloquent areas, lower the chance of being resected surgically. Early detection of these asymptomatic tumors is beneficial, as less surgical removal and manipulation of sensitive brain regions are required [10]. Some experts believe that CNS cancers are incurable because they are diagnosed too late [10]. Early detection efforts for CNS cancers, especially in family members of currently diagnosed cases [11], seems to be promising.

A brain tumor is a costly disease [12]. There seems to be a relation between the socioeconomic status of populations and the epidemiologic indices [13]. Age-standardized death rates and DALYs have been decreasing in high, high-middle, and middle socio-demographic index (SDI) quintiles, while it is the opposite in low SDIs [2], pinpointing the importance of investments in health systems and the need for resource allocation. Most available studies are about the effects of the healthcare system on patients with CNS cancers upper SDI countries. There seems to be a need for investigation in other nations and regions, as well as worldwide surveys to address the burden of CNS cancers in all regions of the world, in addition to the quality, equity, and accessibility to the health services.

The lack of evidence on the health systems’ approaches to confront the CNS cancers’ burden and providing a suitable quality of care for these patients guided us to find a method of examining nations on the effectiveness of care for this neoplasm. In this manuscript, we used the GBD database [14] to evaluate the health systems’ quality of care and inequities, among different nations and regions.

## Material and methods

### Overview

We retrieved primary measures from the GBD 1990-2017 to combine them and calculate the secondary indices that are closely related to the quality of care [visit Supplementary Appendix I (S1) in S1 Appendix for a description of these indices]. The secondary indices were used to estimate a third all-in-one index, Quality of Care Index (QCI), as a proxy of health systems’ overall effectiveness in managing CNS cancers. This study is consistent with GATHER guidelines [15].

### Definitions

#### Quality detection indices

Age-standardized primary measures were acquired from GBD in August 2019 [14] to calculate four subsequent secondary indices. The secondary ratios indirectly show how good health systems care and cover the burden of diseases’ and improve survival [16,17]:

$$Mortality\;to\;incidence\;ratio\;(MIR)\;(x)=\frac{\;Death\;(x)}{Incidence\;(x)}$$

$$DALYs-to-Prevalence\;(x)=\frac{\;DALYs\;(x)}{Prevalence\;(x)}$$

$$Prevalence-to-Incidence\;(x)=\frac{\;Prevalence\;(x)}{Incidence\;(x)}$$

$$Years\;of\;Life\;Lost\;(YLL)-to-Years\;Lived\;with\;Disability\;(YLD)\;(x)=\frac{\;YLL\;(x)}{YLD\;(x)}$$

Where x is the name of the region or country. For ease of interpretation, the secondary ratios were integrated into a 0-100 rescaled single index. The method used to combine these four secondary ratios into a singular index, the Quality of Care index (QCI), is detailed in the Statistical Analysis section and Supplementary Appendix I (S1) in S1 Appendix. Following is the final query of the QCI calculation:

$$PCA_{score}(x)=0.5003587MIR(x)+0.5007964\zeta YLL-to-YLD(x)+0.5002090\zeta DALY-to-Prevalence(x)-0.4986332\zeta Prevalence-to-Incidence(x)$$

ζ: These items should be first transformed to standard (*ζ*) -1 to 1 spectrum by this formula

$$\zeta MIR\;(x)=\frac{\;MIR(x)-\mu }{\sigma }$$

Where x is the data point (e.g. age-standardized MIR of Afghanistan in 2017 for both sexes), μ is the mean, and σ is the standard deviation of the variable in data (e.g. MIR). QCI was retrieved by re-scaling the PCA_score_ into 0-100 spectrum.

$$QCI\;(x)=\frac{\left[PCA_{score}\;(x)–min\;PCA_{score}\right]}{\left[max\;PCA_{score}–min\;PCA_{score}\right)}$$

Higher QCIs (closer to 100) showed a better quality of care. The calculated QCI values for every nation and region were further calculated. SDI is the mean of per capita income, education, and fertility of world nations [18]. Countries were divided into five SDI quintiles based on their 2017 update of GBD. Association of QCI, as a surrogate of care quality with the SDI quintiles was analyzed.

#### Inequity patterns

*Gender disparity*. We separately calculated QCI of males and females and made the female-to-male QCI ratio refereed as the Gender Disparity Ratio (GDR).

$$GDR\;(x)=\frac{\;QCI_{females}\;(x)}{QCI_{males}\;(x)}$$

Where x is the name of a country or region. Values closer to 1 indicated lower gender disparity. Calculated GDRs were then transformed to their subsequent countries on a world map. For a better understanding, we depicted the gender disparity in different ages in global, regional, and SDI scales.

*Age disparity*. QCI for each age group was calculated separately in global, regional, and SDI scales. Ages < 20 years were defined as childhood, 20-65 as adulthood, and > 65 as elderly.

### Data resources

All the estimations were reproduced from the GBD 1990-2017 [14]. Methods used for estimating the primary indices used in this manuscript are detailed elsewhere [2]. In GBD, CNS cancers include International Classification of Diseases (ICD; 10^th^ edition) codes C70.0-C72.9 (C70: malignant neoplasm of meninges; C71: malignant neoplasm of the brain; C72: malignant neoplasm of the spinal cord, cranial nerves, and other parts of the CNS) [2]. Age-standardized measurements were reported for 100,000 persons.

### Statistical analysis

The GBD world population was used to calculate age-standardized values for the primary indices. A 95% uncertainty interval (UI) was reported for primary indices. Results were considered significant if the UI’s of groups of comparison did not overlap. All the statistical analyses and maps in this manuscript were carried out using R statistical packages v3.4.3 (http://www.r-project.org/, RRID: SCR_001905). For a stepwise walkthrough of calculations visit the study protocol [19]. Missing values have been calculated by GBD using specific models [2]. Outliers of QCI and GDR were detected to be interpreted with caution and were not omitted from the analyses.

### Principal component analysis

Principal component analysis (PCA) was utilized to achieve a single surrogate index from a group of variables. PCA uses the above-mentioned secondary measures as entry variables to extract orthogonal or uncorrelated components/linear combinations [20]. The first component with maximum correlation with entry variables and variance was the principal component, that is named QCI in this manuscript. Further details of steps and mathematical calculations are accessible from the study protocol [19] and Supplementary Appendix I (S1) in S1 Appendix.

### Validity of QCI

Healthcare Access and Quality (HAQ) index has been previously introduced by IHME as a proxy of care quality and access [21]. A mixed-effect model was applied by considering QCI as a dependent variable and inpatient healthcare utilization, outpatient healthcare utilization, brain and other CNS cancers death, and prevalence as independent variables by random effects of countries. We utilized age-standardized values to enable comparison. This comparison was aimed to investigate the validity of QCI to measure the quality of care and access. We calculated our index’s correlation with the HAQ index to be 0.74.

## Results

### Overview

In 2017, there were 405,000 new incident cases (95% UI 351,000 to 442,000; Age-standardized rate of 5.16 in 100,000 person-year [4.46 to 5.63]) and 247,000 deaths (212,000 to 265,000; 3.11 [2.68 to 3.34]), globally (Table 1). It is estimated that about 8,744,000 DALYs (7,652,000 to 9,554,000; 122.57 [95.69 to 151.42]) were attributed to CNS cancers in that year, which 57.47% of these DALYs were directed to the male population. It is estimated that the incidence of CNS cancers has significantly increased in the last 27 years. It is shown that incidence of CNS cancers increases as the economic features of nations improve (higher SDI regions). Interestingly, age-standardized death rates and DALYs are increasing in wealthy countries (Supplementary Appendix II (S2) Tables 1 & 2 in S2 Appendix). All GBD data used in this manuscript (incidence, prevalence, mortality rate, DALY, years of life lost (YLL), and years lived with disability (YLD)) are available in the GBD Compare and S2 Tables in S2 Appendix.

**Table 1:** Global crude and age-standardized prevalence, incidence, DALYs, YLLs, and YLDs for CNS cancers in 2017 and 1990.

	Prevalence		Incidence		Death		DALY		YLD		YLL	
	Count	Rate	Count	Rate	Count	Rate	Count	Rate	Count	Rate	Count	Rate
1990 estimate (95% UI)	630,000 (512,000 to 762,000)	12.35 (10.28 to 14.76)	194,000 (159,000 to 233,000)	3.97 (3.32 to 4.71)	142,000 (117,000 to 170,000)	3.03 (2.56 to 3.58)	6,368,000 (4,836,000 to 7,987,000)	111.91 (97.82 to 122.53)	71,000 (48,000 to 95,000)	1.43 (0.99 to 1.91)	6,297,000 (4,770,000 to 7,914,000)	121.13 (94.53 to 149.67)
2017 estimate (95% UI)	1,700,000 (1,470,000 to 1,890,000)	22.01 (18.96 to 24.47)	405,000 (351,000 to 442,000)	5.16 (4.46 to 5.63)	247,000 (212,000 to 265,000)	3.11 (2.68 to 3.34)	8,744,000 (7,652,000 to 9,554,000)	122.57 (95.69 to 151.42)	167,000 (117,000 to 223,000)	2.13 (1.49 to 2.85)	8,577,000 (7,527,000 to 9,359,000)	109.78 (96.10 to 120.04)
Statistically Significant?	*	*	*		*				*			

DALY: Disability-adjusted life years; YLL: Years of life lost; YLD: Years lived with disability; UI: Uncertainty interval.

### QCI

**Fig 1:**
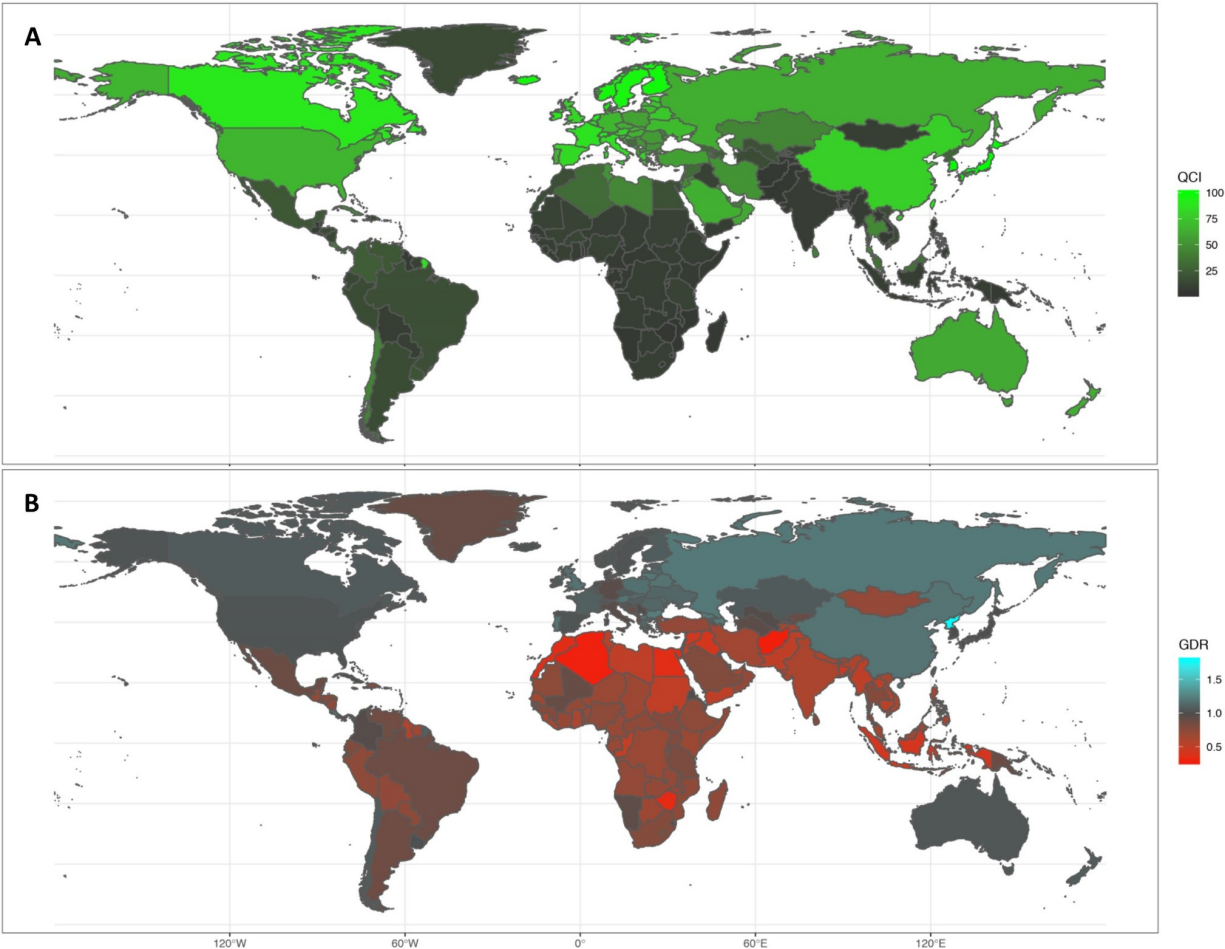
A) Quality of Care Index (QCI) map for CNS cancers in both sexes in 2017 B) Sexual disparity in different nations depicted from the gender disparity ratio (GDR) in 2017.

Globally, QCI in 1990 has been calculated to be 27.4, while in 2017 it was 55.0. Considering the SDI quintiles in 2017, high SDI and low SDI countries with a QCI of 82.4 and 7.7 were the two extremes of health-care quality in the case of CNS cancers, respectively. Regionally, in 2017, the Western Pacific Region (QCI of 76.9) followed by the European Region (72.5) and the Region of the Americas (47.4) were the 3 regions with the highest healthcare qualities. In contrast, the African Region (9.9), South-East Asia Region (10.3), and Eastern Mediterranean Region (25.6) were on the other side of this spectrum. Considering countries, Singapore with a QCI of 100 was the ideal country referencing this lethal cancer in 2017, followed by Japan (99.9) and South Korea (98.9). In contrast, Swaziland (2.5), Lesotho (3.5), and Vanuatu (3.9) were countries with the poorest conditions considering QCI for CNS cancers (Fig 1A; S2 Table 3 in S2 Appendix).

### Age and gender inequity

**Fig 2:**
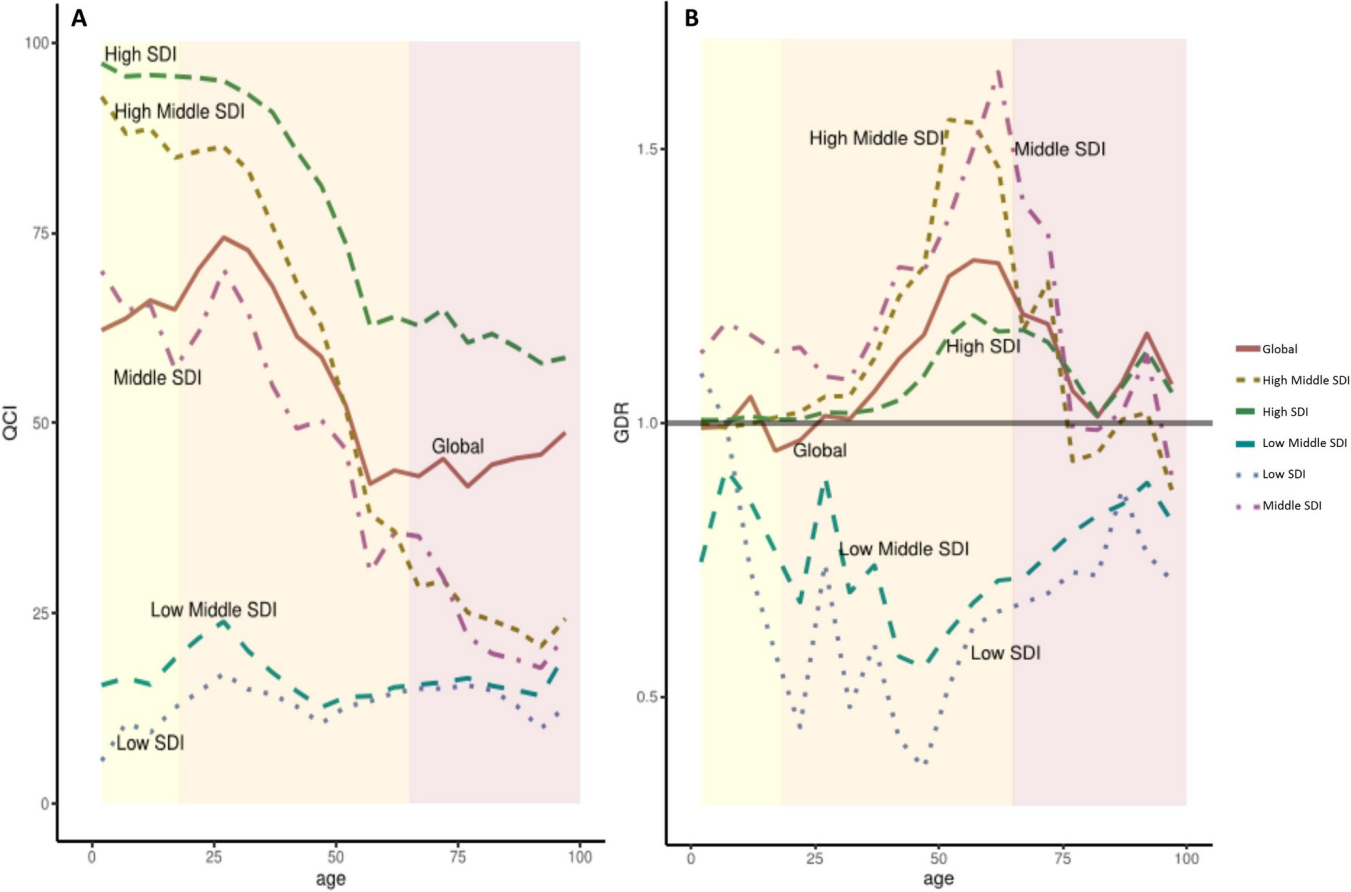
Disparity patterns in global and socio-demographic index (SDI) quintile regions. A) Age disparity regarding access to health services estimated from the Quality of Care Index (QCI). B) Gender disparity ratio (GDR) in different age groups. Yellow: childhood and adolescence group, aging less than or equal to 18. Orange: adulthood group, ages between 18 and 65. Pink: elderly group, ages more than 65.

Gender and age equity in quality of care are the mainstay of health systems standards. In 2017, a serious age disparity concerning CNS cancers’ care quality had been detected based on GBD. Globally, the best care was given to patients in their early adulthood [Fig 2A; QCI: all ages overall 55.04, childhood (range 62.18-70.31), adulthood (43.00-74.35), elderly (41.63-48.73)]. A similar trend found to exist in middle SDI countries [QCI: all ages overall 48.2, childhood (57.1-69.9), adulthood (30.3-70.1), elderly (17.8-29.6)]. In upper SDI quintile countries, pediatric patients had better quality of care; while their geriatric population received no good care [QCI: all ages overall (high SDI: 82.4; high-middle SDI: 69.1), childhood (95.3-97.2; 85.7-92.8), adulthood (62.8-94.9; 28.4-86.2), elderly (57.8-64.9; 20.6-29.3)]. In low and low-middle SDI countries quality of care for CNS cancers was poor for all ages, however, children faced critically worse condition [QCI: all ages overall (low-middle SDI: 10.9; low SDI: 7.7), childhood (15.5-21.7; 5.5-14.6), adulthood (12.6-23.8; 10.4-17.0), elderly (14.1-19.6; 9.8-15.0)].

As is evident from GDR maps (Fig 1B), gender disparity existed in many regions of the world with different economic conditions. Globally, it seems that females (in all age groups combined) had better CNS cancers care quality [GDR: all ages overall 1.08, childhood range 0.94-1.04, adulthood 1.00-1.29, elderly 1.05-1.18] (Fig 2B). The pattern of this disparity changed as the socio-demographic position of countries advanced. In lower SDI countries, GDR values were (in almost all ages) less than 1 [GDR: all ages overall (low-middle SDI: 0.70; low SDI: 0.59), childhood (0.67-0.91; 0.44-1.08), adulthood (0.55-90; 0.36-0.74), elderly (0.75-0.81; 0.67-0.87)], indicating a superiority of males to females. On the other hand, the high SDI nations harbored GDRs closer to 1 compared to other nations [GDR: all ages overall 1.05; childhood 0.67-0.91, adulthood 0.55-90, elderly 0.75-0.81]. As a matter of fact, in high-middle and middle SDI countries, the huge disparity was found in favor of females during adulthood, while fading in both childhood and elderly [GDR: all ages overall (high-middle SDI: 1.09; middle SDI: 1.30), childhood (0.44-1.08; 1.12-1.18), adulthood (0.36-0.74; 1.07-1.64), elderly (0.67-0.87; 0.90-1.34)]. North Korea with a GDR value of 1.78 was three standard deviations higher from the mean GDR values of the rest of the countries and found to be an outlier. [For GDR values of other nations, gender-specific QCIs, and values of past years visit S2 Table 3 in S2 Appendix].

## Discussion

The findings of this manuscript mainly indicate the existing disparity concerning CNS cancers quality of care, in 2017. Due to the rising burden of CNS cancers worldwide, most of the wealthy regions of the world provided an acceptable care to patients with brain tumors. But it should be reminded there are still neglected sub-populations, such as mid-aged adults (40-65) and elderly (>70) in wealthy nations. Though there was lower overall QCI in poorer regions of the world, females are believed to receive lower quality services.

Our results showed that the burden of CNS cancers ⎯ evident from the crude increase in prevalence, incidence, DALY, YLL, and YLD (not significant for DALY and YLL) measures ⎯ has increased between 1990 and 2017. Moreover, some of the age-standardized indicators of disease burden showed a decreasing pattern, due to improved given care and diagnostic approaches. These findings are consistent with prior efforts to highlight the burden of CNS cancers [2,22]. As the socio-demographic position of nations heightened, the incidence rate also increased. This observation mostly is due to more efficient screening and diagnosis approaches in such countries. The global age-standardized incidence rate of CNS cancers has been increasing almost linearly in the last 27 years, except for the 1999-2006 period. This pattern of change was also evident in other indicators of burden. Prevalence and YLD were increasing roughly linearly in the last 27 years. In other words, age-standardized YLL, though being ascending before 1995, decreased abruptly to the value of about 110 years for 100,000 population in 2006 and stayed the same to this date. All taken together, 122.57 age-standardized DALYs have been estimated to be attributed to CNS cancers in 2017 globally. This value has shown an overall decreasing pattern in the last 27 years of GBD; while it was increasing in the 90s, decreasing after that and remaining around 120 in the last ten years. This trend was also replicated in previous surveys [22–25]. DALY has followed a similar pattern as YLL; while the YLD has remained relatively the same. This finding indicates that while health systems were successful in decreasing the mortality of CNS cancers, the disabilities appointed to a condition like brain tumors have remained unchanged and efforts are needed to enhance survivors’ lives.

Globally, QCI is calculated to be 55.0, indicating that care given to the global population is almost half of the best health systems being in action, Singapore, Japan, and South Korea. Moreover, two high-income regions (Western Pacific and European) had QCIs greater than global value, while other regions of the world with lower monetary potency had worse condition (African, South-East Asia, and Eastern Mediterranean Regions). This finding points to the notion that care quality requires great resource allocation [12]. As the burden on health systems is transitioning from communicable diseases toward non-communicable diseases, especially in developing countries, it is anticipated that the gap between regions will become more pronounced [26,27]. CNS cancers usually are considered when focal neurologic symptoms emerge, not in routine general clinic visits. Lack of reliable and available screening methods, in conjunction with costly diagnostic tools (e.g. MRI) and treatment techniques (like surgery, gamma-knife, and chemotherapy) make CNS cancers extreme supply consumers [28]. Although lead time bias might convey confounding effects of early detection on quality assessment efforts (i.e. QCI) in regions where screening methods and more dedicated health systems are in action.

CNS cancers are among the most prevalent solid tumors of the human body [29]. It is believed that CNS cancers and other neurosurgical conditions happen more in males [2,25,30,31]; although some surveys had claimed otherwise [32]. Gender disparity exists nowadays when we inspect access to health systems and suitable care [33]. Results of the current study show that globally females have better care access. This condition can be due to more genuinely seeking health services and treatments by women [34,35], lower overall life expectancy than males as they are more prone to death due to cardiovascular events and injuries [36,37] and as a result mortality (as an indicator of quality and component of QCI) due to chronic diseases such as CNS cancers is expected less in males, affirmative actions in developed and rapidly developing countries toward females empowerment and independence [38], and inherent differences between males and females [39,40]. However, gender disparity is more pronounced in low and low-middle SDI countries; a huge disparity exists in favor of males. The male predominance being in action in such countries withholds females from access to costly CNS cancer treatments [41]. On the contrary, among developing countries of middle and high-middle SDI countries, females are given better care, partly due to the abovementioned reasons. Interestingly, in the uppermost SDI group of nations, this gender disparity almost resolves. Age-groups’ equity in access to care is as important as gender equity. Elderly populations have not given proper care regarding CNS cancers [42,43]. This finding can be due to the lower life expectancy in older ages, which makes it not logical to make them tolerate the heavy load of invasive treatments coming with CNS cancers besides the governmental rationale of cost-beneficial resource allotment [44]. In lower-wage countries, the age disparity is not that intensive, while QCI is demandingly low for all. Gender disparity during childhood and adolescence is not an issue in most regions of the world; except for low SDI countries. In such regions, male progenies have better healthcare quality.

Study on risk factors associated with a disease is fundamentally important as both population- and individual-level interventions for controlling them can influence the burden of that certain disease. While many efforts are taken to understand the risk factors of CNS cancers, results are not very conclusive [11]. Besides genetic/familial factors and environmental measures are not fully proven to be associative. Ionizing radiations, especially head computed tomography (CT) scans during childhood [45], are the most notified risk factor of CNS cancers. No occupational or non-ionizing radiation exposure, such as magnetic fields and cell phones, have had significance more than a guess. Interestingly, a history of allergies and atopies may associate with a lower rate of CNS cancers. Although it’s more of an epidemiologic premise, biological attributes have not been addressed yet. To the current date, other than limiting and substituting the ionizing CT scans of head, there is no rationale for intervening in the risk factors with the aim of preventing CNS cancers.

Providing acceptable quality of care is fundamental to universal health coverage. The global ambition toward global health coverage can’t be reached unless proper measures be available to justify the current status and pace of healthcare quality and access. A similar effort has been carried out before to indirectly assess such measure by transforming MIR of different cancers into a single 0-100 index as Healthcare Access and Quality (HAQ) index [21]. Death is the end component of cancers and decreasing the death rate is basically the main goal of health systems, although there are major drawbacks in this path. Quality care not only decreases the mortality rate of cancers but also extends the life length (represented in decreasing YLL), improves the life quality (represented in decreased DALY and YLD), and leads to more people living with the disease (represented in the prevalence of disease). QCI utilizes more components into account than HAQ and also we found a strong correlation between these two, indicating that both are conferring similar annotation. These metrics, regardless of their methodological differences, are correlated with a total expenditure of a given health system, exemplifying a single notion: allotment of resources increases the overall quality of care of brain and other CNS cancers.

The main limitation of QCI is that it is restricted to the only disease that is being calculated for, for example CNS cancers. QCIs of different diseases cannot be compared to each other. Racial disparity and inequity are fundamental throwbacks of nowadays health systems [46]. GBD has not yet stratified its population and estimates based on human races and ethnicities. Although using available results about gender and age groups provided insight about the gender and age disparity. Lead time bias has confounding effects on QCI estimate of regions pertaining to screening methods due to early detection of conditions. We tried to present the latest update on the CNS cancers epidemiologic indices and highlight the rising burden of such disease. A new approach and cumulative index for evaluation of the quality of care and treatment access has been proposed in this work. The scarceness of current knowledge on the risk factors of CNS cancers and unavailability of the history of ionizing radiation of head in GBD data source withdrew us from adjusting QCI for risk factors. Findings and analyses of this study can be used for strategic planning and resource allocation.

## Conclusion

CNS cancers are responsible for a major mortality and morbidity globally and their burden is increasing steeply. High and high-middle income regions have the most suitable and efficient policies to confront the rising burden of CNS cancers. Future studies are needed to investigate high-middle countries’ health systems with their adopted legislations and programs, such as Singapore and Japan.

## Supporting information

S1 Appendix Details of secondary indices and mathematical calculation of QCI. (DOCX)

S2 Appendix Details of primary indices, QCI, and GDR by globe, regions, and countries in 1997, 2007, and 2017. (XLSX)
