# Supplementary material for: A global, regional, and national survey on burden and Quality of Care Index (QCI) of brain and other central nervous system cancers; global burden of disease systematic analysis 1990-2017
Source: PLoS One. 2021 Feb 22;16(2):e0247120. doi: 10.1371/journal.pone.0247120 (PMC7899371; doi:10.1371/journal.pone.0247120)
Supplement: S1 Appendix — (DOCX) [file pone.0247120.s001.docx]

**Supplementary Appendix I:**

**Secondary indices**

1. Mortality-to-incidence ratio (MIR):

$$MIR (x)= \frac{Death (x)}{Incidence (x)}$$

This ratio takes into account that considering a stable incidence (denominator) of CNS cancers in a geographical region, higher mortality values pertain to worse care provided to these patients. It is believed that whenever a new brain/cord tumor case emerges in a population, averting his/her death is a promise of the health system.

1. DALY-to-prevalence ratio:

$$DALYs-to-Prevalence (x)= \frac{DALYs (x)}{Prevalence (x)}$$

Similarly, this ratio indicates that in cases of similar prevalence of CNS cancers in different regions, higher DALY is indexed by worse care quality.

1. Prevalence-to-incidence ratio:

$$Prevalence-to-Incidence (x)= \frac{Prevalence (x)}{Incidence (x)}$$

This ratio combines prevalence and incidence values. To better understand the definition of this ratio, consider occasions with similar incidence rates of this form of cancers, higher prevalence of it premise that patients are managed more accordingly and their deaths are prevented.

1. YLL-to-YLD ratio:

$$YLL-to-YLD (x)= \frac{YLL (x)}{YLD (x)}$$

Poor health quality in a region results in higher YLLs and less YLDs (patients are ceased earlier than their region mean expected life years). This ratio assumes that living with disability related to CNS cancers is superior to dying in advance. This ratio also highlights the effectiveness of the health system to post-pone patients deaths. Higher values represent worse conditions.

**Quality of Care index (QCI)**

PCA is a mathematical approach that uses a n-dimensional transformation of data points to achieve an eigenvector that resembles and covers the largest span of data on its axis. In our case, we had four secondary measures (i.e. MIR) that we wanted to composite into one characteristic; so it is a four-dimension contemplate with +200 location data x in 27 years. After running the PCA code on this dataset, we investigate the amount of variance among data points that can be explained by different eigenvectors (components). Conventionally, the one vector that has the most discrimination ability (explaining the largest amount of variability and variance of data points) is defined as the first component of PCA and is considered as a composite characteristic. This approach was then reiterated for each of age-group x sex-groups. The PCA scores (=loading factors) were used and rescaled into 0-100 and named QCI. We used a post-hoc investigation on QCI calculations and it has grabbed 97.3% of variation [in both-sex age-standardized population calculation] of the four input parameters. On Average, PCA has retrieved 95.5% of variation in calculation for different subpopulations (consisting of different combination of sex-groups and age-groups) and ranging between 87.4% and 98.7%. Below figures show the properties of PCA calculation. Also, checked for correlation of QCI and each of the four entry variables, all |correlation coefficients| were greater than 0.98. Appropriate correlation of QCI with Healthcare Access and Quality (HAQ) index [as an approved index 一 0.74 correlation with age-standardized both-sex QCI] and its large amount of grabbed variability and association with either of input variables made us confident that QCI can be an acceptable indicator of quality in CNS cancers.


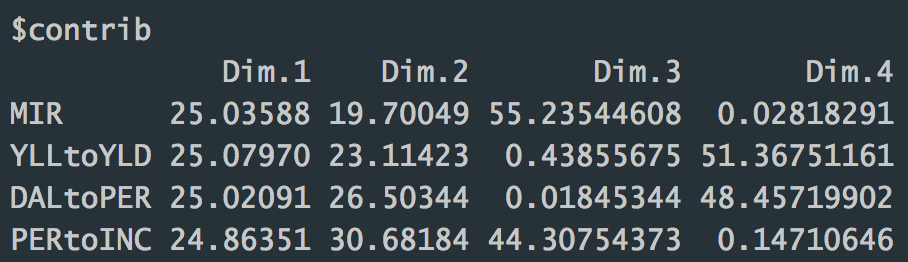


Figure 1. Percent of contribution of each of four secondary variables (as entries) with different components [=dimensions] of PCA on age-standardized both-sex subpopulation of CNS cancers from 1990 to 2017.


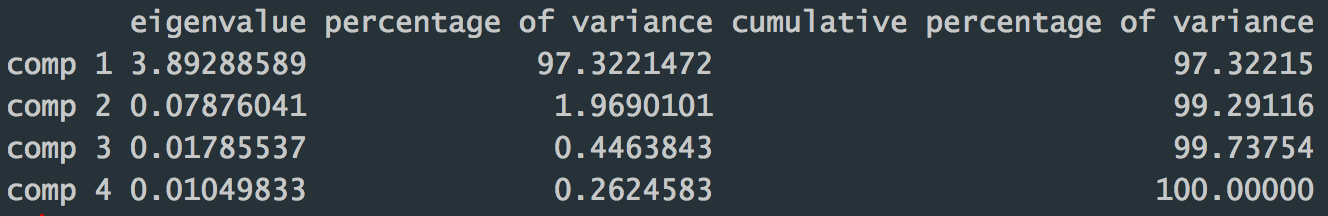


Figure 2. The eigenvalue of the first component was close to 4 (as the largest possibility in a 4-dimension PCA) and has grabbed 97.3% of variability and information of total data points (20,412 points) in age-standardized both-sex population. Also, it revealed a mean variance of 95.5% on average in different subpopulations (cumulatively, 12,447,288 data points).


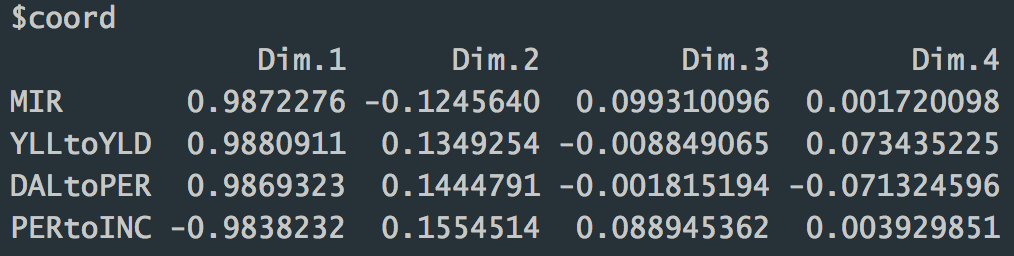


Figure 3. The slopes of each of four entry variables in each of dimensions. It is worth noting the closeness of slopes to 1 (as the perfect component) in the dimension #1 (first component).


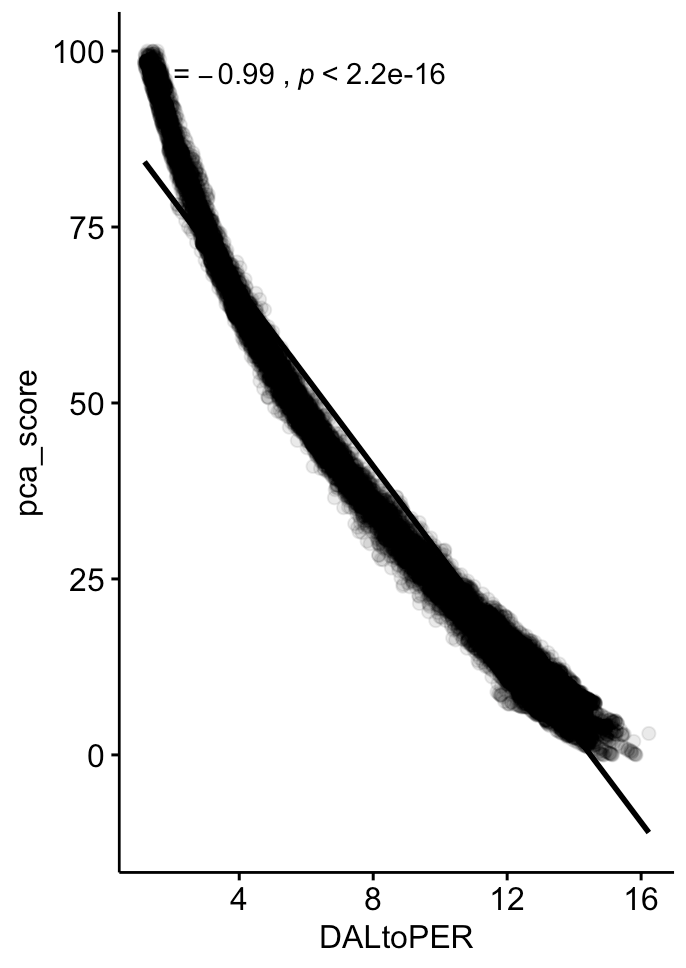

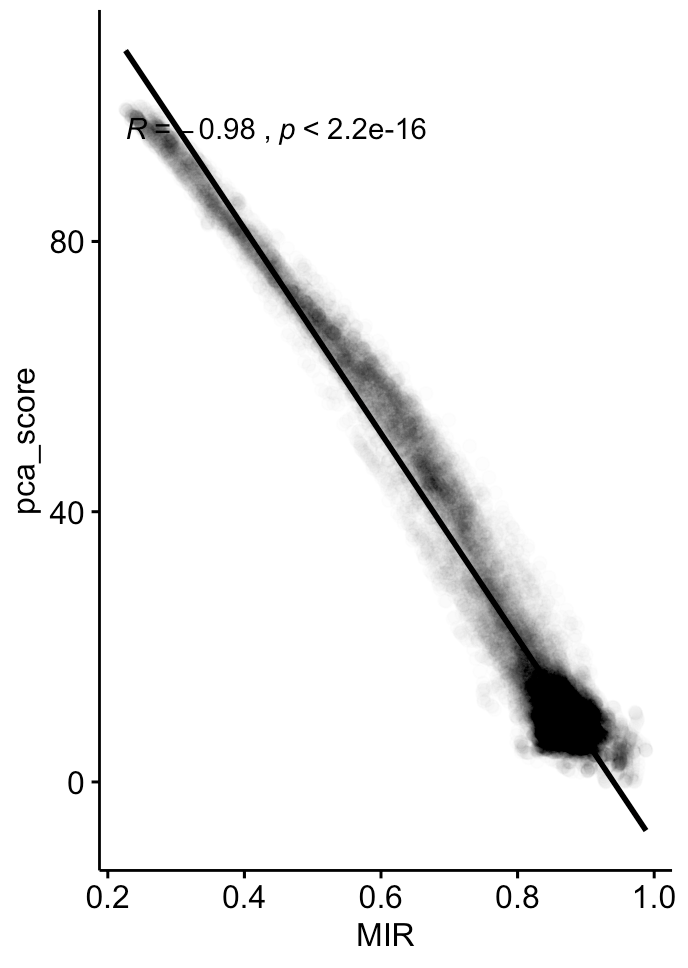

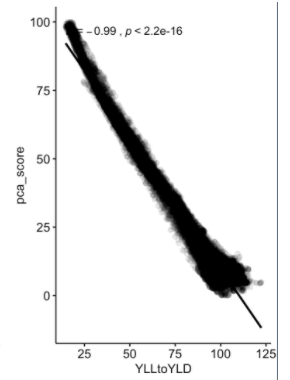

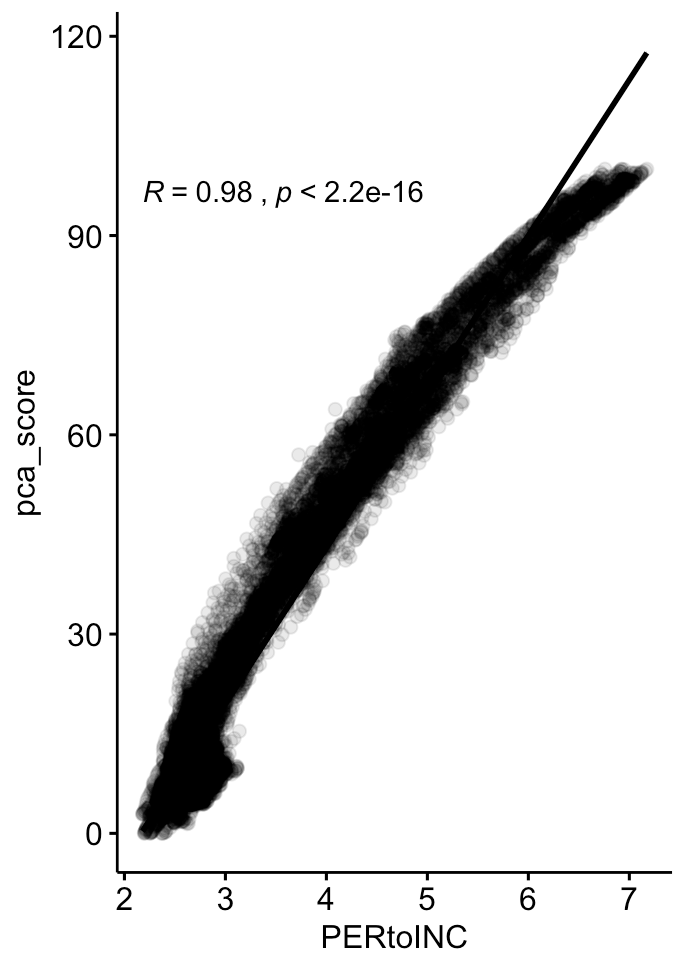


Figure 4. The correlation of each of entry variables with QCI. Prevalence-to-incidence had an inverse relation as it was anticipated. It should be reminded that in case of constant incidence, regions with higher prevalence rate have provided better care to their population.


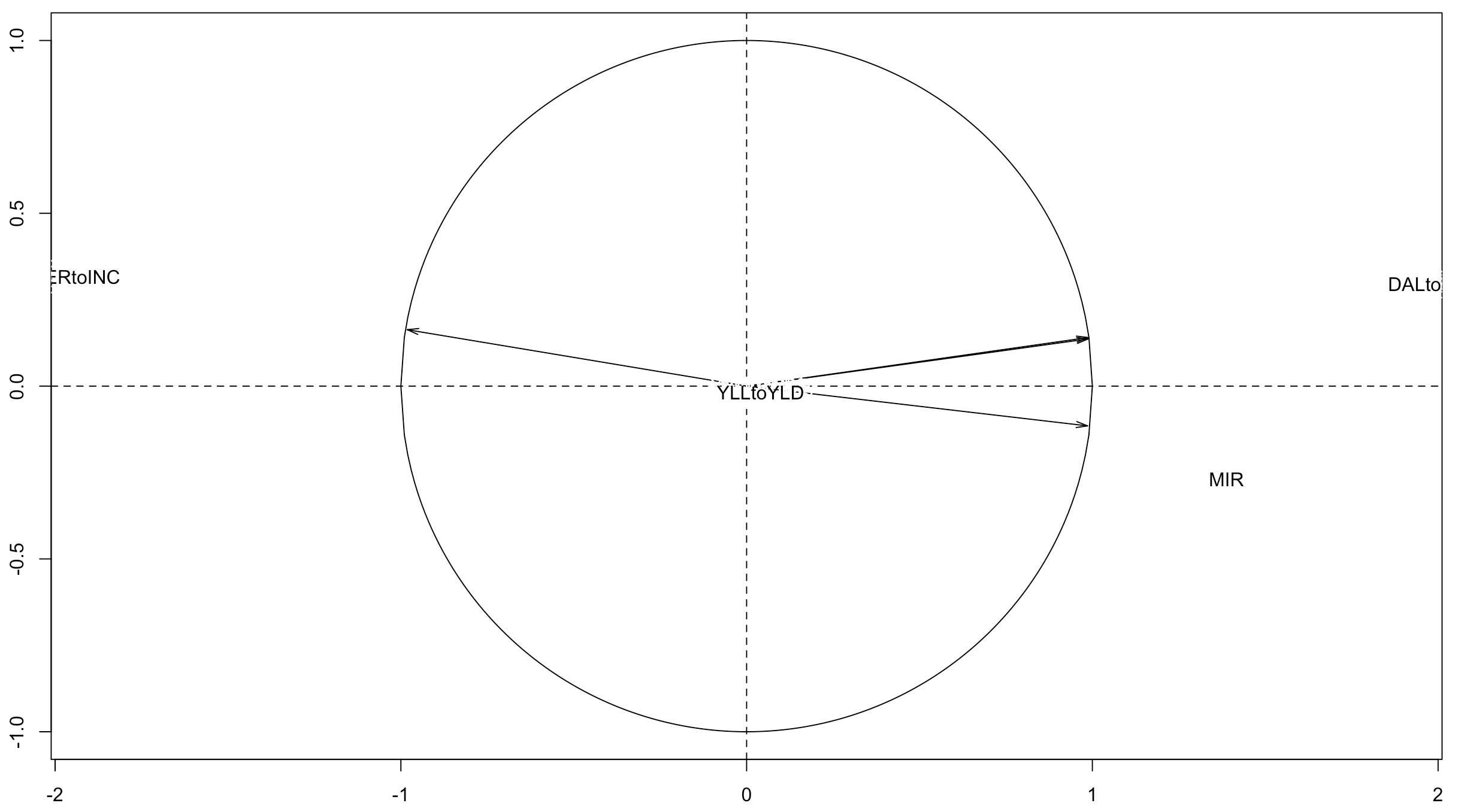


Figure 5. The orientation of each of four variables on the transformed coordinate system. The x-axis represents the 1st component and the y-axis as the 2nd component. The orientations come from the coordinates and slopes of each variable on that particular dimension. [DALY-to-prevalence and YLL-to-YLD vectors have lied on exact same directions]. Prevalence-to-incidence presented a reverse orientation to others.


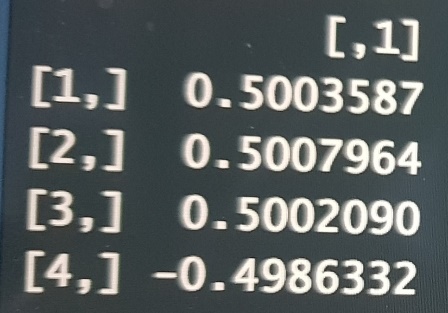


Figure 6. The loading factors (β) of each of entry variables regarding first component.

Following is the final query of QCI calculation:

$$PCA_{score}\left( x \right)= 0.5003587MIR\left( x \right) + 0.5007964\zeta YLL-to-YLD \left( x \right) + 0.5002090\zeta DALY-to-Prevalence(x) - 0.4986332\zeta Prevalence-to-Incidence(x)$$

ζ : These items should be first transformed to standard ($\zeta$) -1 to 1 spectrum by this formula

$$\zeta MIR (x)= \frac{MIR\left( x \right) - \mu}{\sigma}$$

where x is the data point (i.e. MIR of Afghanistan in 2017 for both-sex and age-standardized all age group), μ as the mean and σ as the standard deviation of that variable in our data (i.e. MIR). QCI will be retrieved by re-scaling PCA_score_ into 0-100 spectrum

$$QCI \left( x \right)= \frac{[PCA_{score} (x) - min PCA_{score} ]}{[max PCA_{score} - min PCA_{score} )}$$
